# Supplementary material for: Time-course swRNA-seq uncovers a hierarchical gene regulatory network in controlling the response-repair-remodeling after wounding
Source: Commun Biol. 2024 Jun 6;7:694. doi: 10.1038/s42003-024-06352-w (PMC11156874; doi:10.1038/s42003-024-06352-w)
Supplement: Supplementary file 2 — Supplementary Information [file 42003_2024_6352_MOESM2_ESM.pdf]

Supplementary Materials for

Time-course swRNA-seq Uncovers a Hierarchical Gene Regulatory Network in Controlling the Response-Repair-Remodeling after Wounding

Xinghai Yu[1, †], Jinghua Zhou[2, †], Wenkai Ye[2, †], Jingxiu Xu[2], Rui Li[3], Li Huang[1], Yi Chai[4], Miaomiao Wen[1], Suhong Xu[2, 4, *], and Yu Zhou[1, 5, 6, 7, *]

This supplementary file includes 11 supplementary figures.

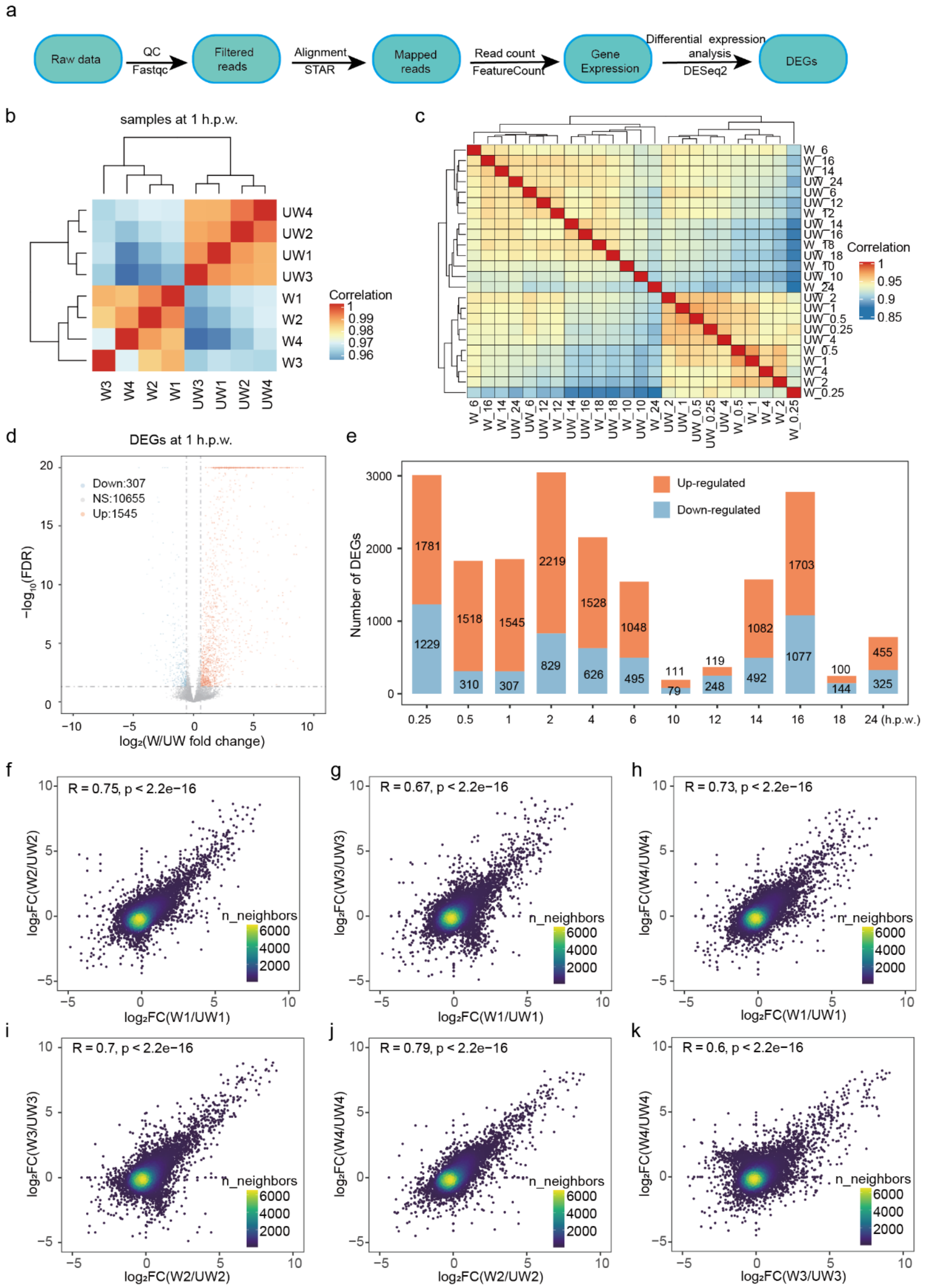

Figure S1. Processing and quality control of time-course swRNA-seq data, Related to Figure 1.

(**a**) Flow chart of the analysis of single nematode RNA-seq. The blue boxes represent the input or output of each analysis step. The analysis steps and the used softwares are described above and below the arrows, respectively. (**b**) Heatmap of correlations

between wound and unwounded samples at 1 h.p.w. (**c**) Heatmap of correlations between different time points. (**d**) Volcano plots show the distribution of differentially expressed genes 1 hour after wounding. Red and blue represent up-regulated and down-regulated genes, respectively. The criteria for differentially expressed genes were a fold-change greater than 1.5 and a calibrated p-value less than 0.05. (**e**) Histogram showing the number of differentially expressed genes at different time points after wound. The red and blue bars represent the number of up-regulated and down-regulated genes, respectively. (**f-k**) Density scatter plot showing the correlation of fold changes(W/UW) in expression between replicates for the 10,500 expressed genes at 1 h.p.w.

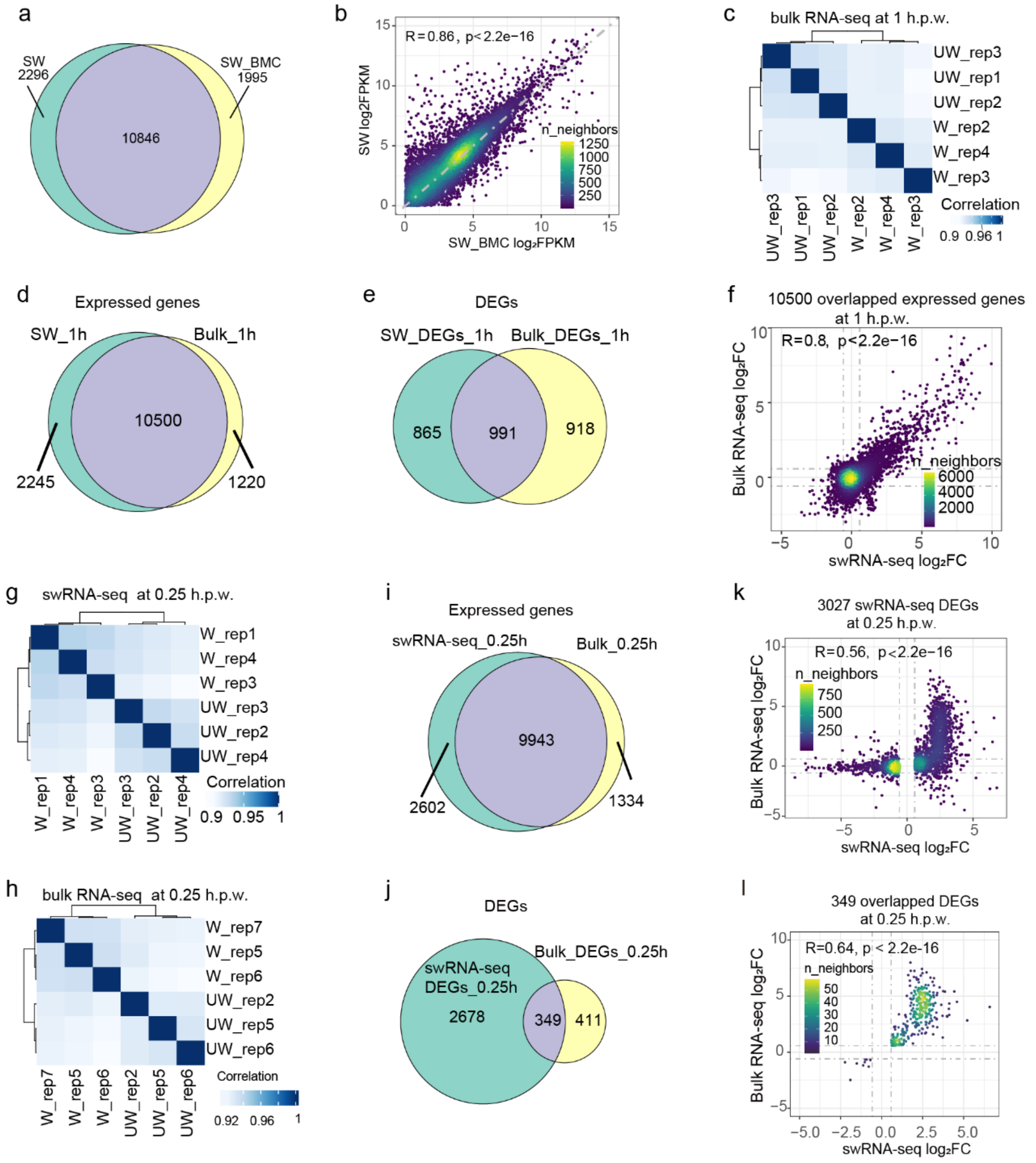

Figure S2. Comparison of our swRNA-seq with other RNA-seq data. Related to Figure 1. (**a**) Venn diagram showing the commonly expressed genes in our swRNA-seq (SW) and the swRNA-seq from a *BMC Genomics* paper (SW_BMC). Expressed genes were defined by requiring the average FPKM > 1 for replicates. (**b**) Density scatter plot showing the FPKM correlation of all commonly expressed genes between SW and SW_BMC. (**c**) Heatmap of correlations between wounded and unwounded samples of bulk RNA-seq at 1 h.p.w. (**d-e**) Venn diagram showing overlap of expressed genes (**d**) and DEGs (**e**) in bulk RNA-seq and swRNA-seq at 1 h.p.w. (**f**) Density scatter plot showing the correlation of the 10, 500 overlapped expressed genes between bulk RNA-seq and swRNA-seq at 1 h.p.w. (**g-h**) Heatmap of correlations between wounded and unwounded samples of swRNA-seq (**g**) and bulk RNA-seq (**h**) at 0.25 h.p.w. (**i-j**)

Venn diagram showing overlap of expressed genes (**i**) and DEGs (**j**) in bulk RNA-seq and swRNA-seq at 0.25 h.p.w. (**k-l**) Density scatter plot showing the correlation of gene expression changes of swRNA-seq DEGs (**k**) and common DEGs between bulk RNA-seq and swRNA-seq (**l**) at 0.25 h.p.w.

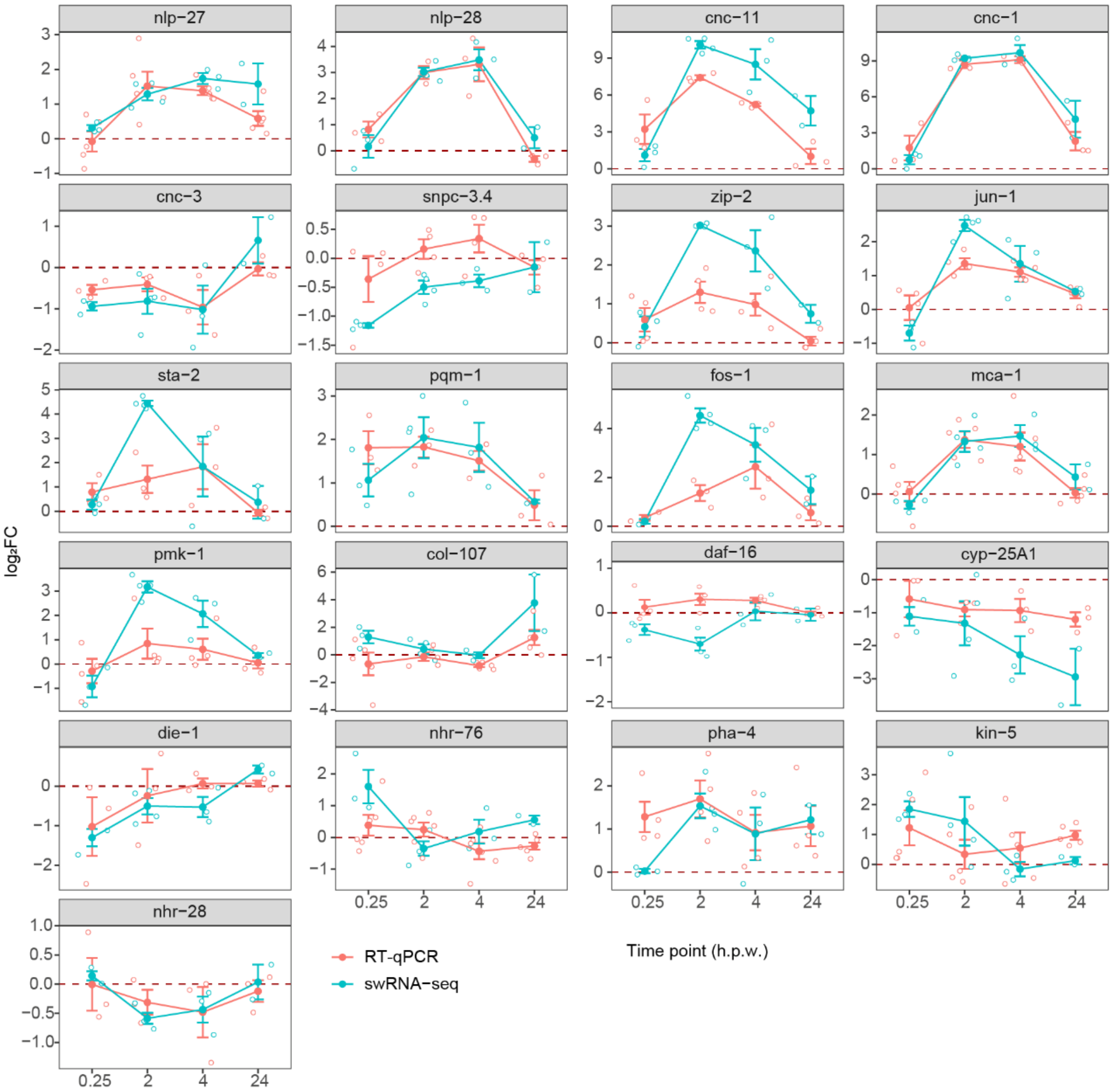

Figure S3. RT-qPCR validation of representative genes in swRNA-seq data. Related to Figure 1. Expression changes at four time points (0.25, 2, 4, 24 h.p.w.) were validated through RT-qPCR. The blue line represents the trend of swRNA-seq and the red line represents the trend identified in RT-qPCR post wounding. The error bar represents the standard error (S.E.) in RT-qPCR measurements.

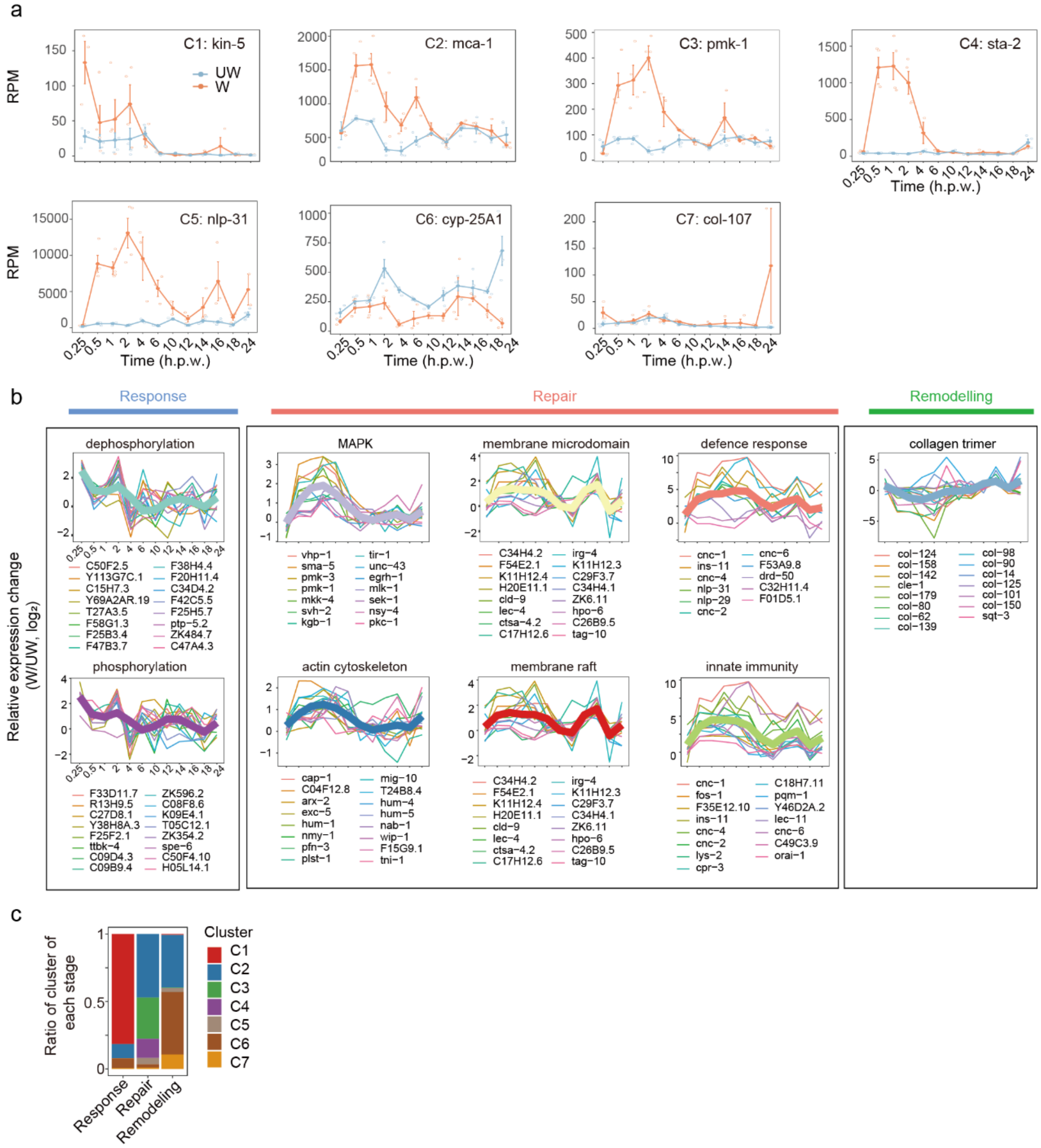

Figure S4. Representative gene examples of dynamic expression changes during wound repair, Related to Figure 2.

(**a**) Dynamic expression profiles of representative genes in clusters 1-7 under unwounded (UW) and wounded (W) conditions. (**b**) Representative GO-terms enriched in the three stages (Response, Repair, and Remodeling) of wound repair and associated genes with their expression profiles. The bold lines represent averaged profiles of corresponding genes shown as thinner colored lines. (**c**) The composition of the 7 clusters (C1-C7) of hiDEGs in the three stages (Response, Repair, and Remodeling) of wound repair.

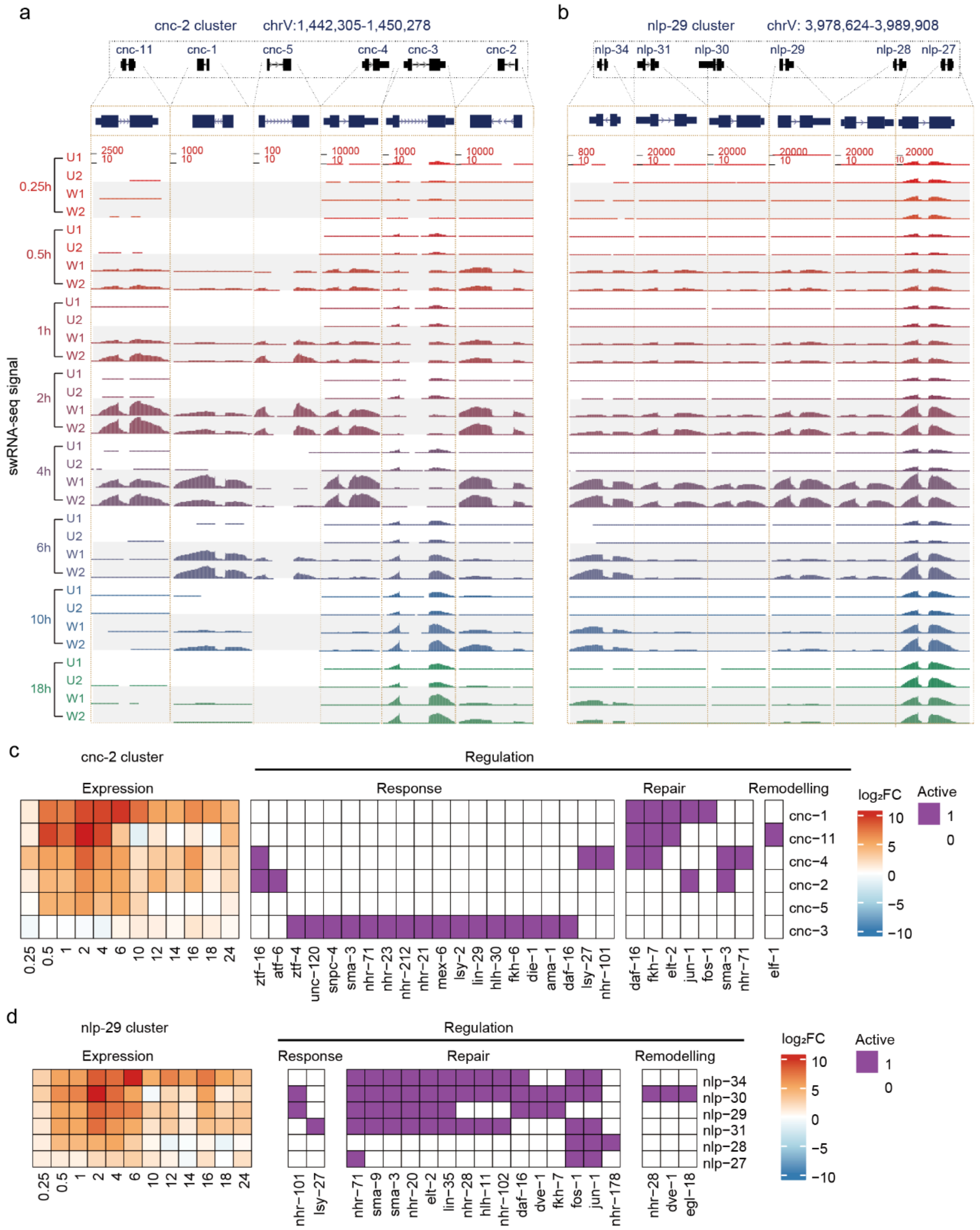

Figure S5. Gene expression changes of two gene clusters, Related to Figure 2. (**a-b**) UCSC genome browser view showing the swRNA-seq signals in the cnc gene cluster (**a**), and the nlp gene cluster (**b**) at different time points under unwounded (replicates: U1, U2) and wounded (replicates: W1, W2) conditions. (**c-d**) Heatmap showing the relative fold-changes between unwounded and wounded conditions for the individual genes in the cnc cluster (**c**) and nlp cluster (**d**).

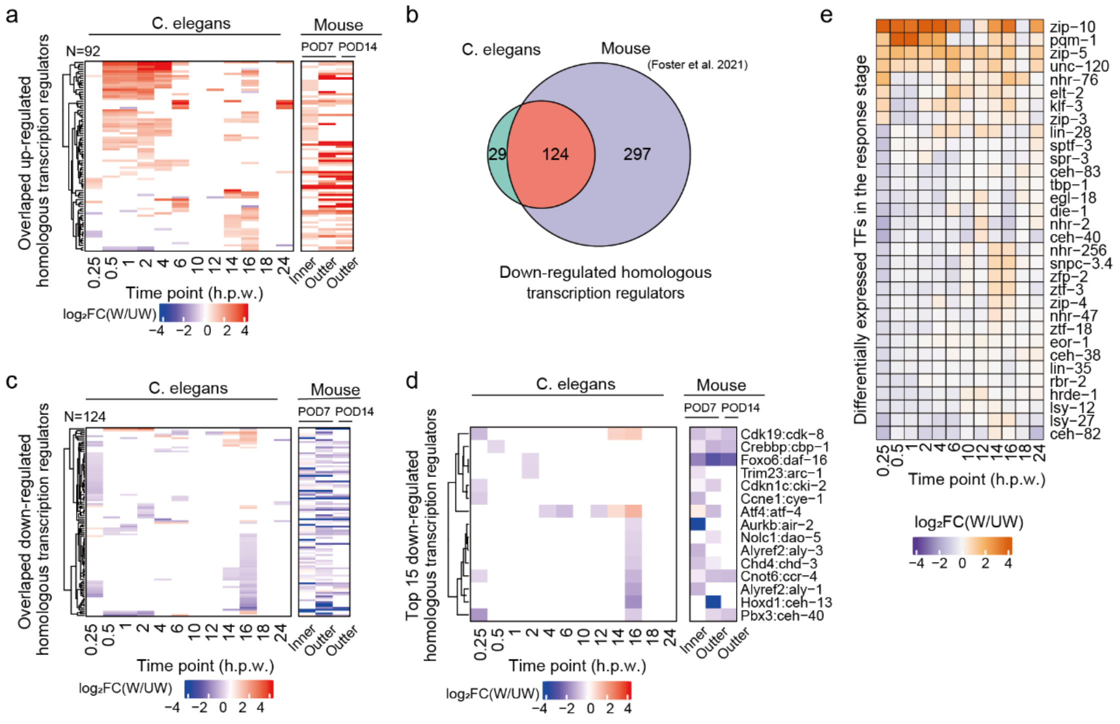

Figure S6. Differentially expressed TFs during wound repair, Related to Figure 3. (**a**) Heatmap showing gene expression changes for the overlapped up-regulated homologous transcription regulators in mouse and *C. elegans*. The mouse data were from Foster et al. 2021. The expression changes were calculated as the $\log_2$ ratio of W (wound) to UW (unwound) conditions from *C. elegans* or mouse. (**b**) Venn diagram showing the overlap of down-regulated homologous transcription regulators in mouse and *C. elegans* after epidermal wounding. (**c**) Heatmap showing gene expression changes for the overlapped down-regulated homologous transcription regulators in mouse and *C. elegans*. (**d**) Heatmap of the top 15 transcription regulators as in **c**. (**e**) Heatmap showing gene expression changes for the significantly differentially expressed TFs at the response stage.

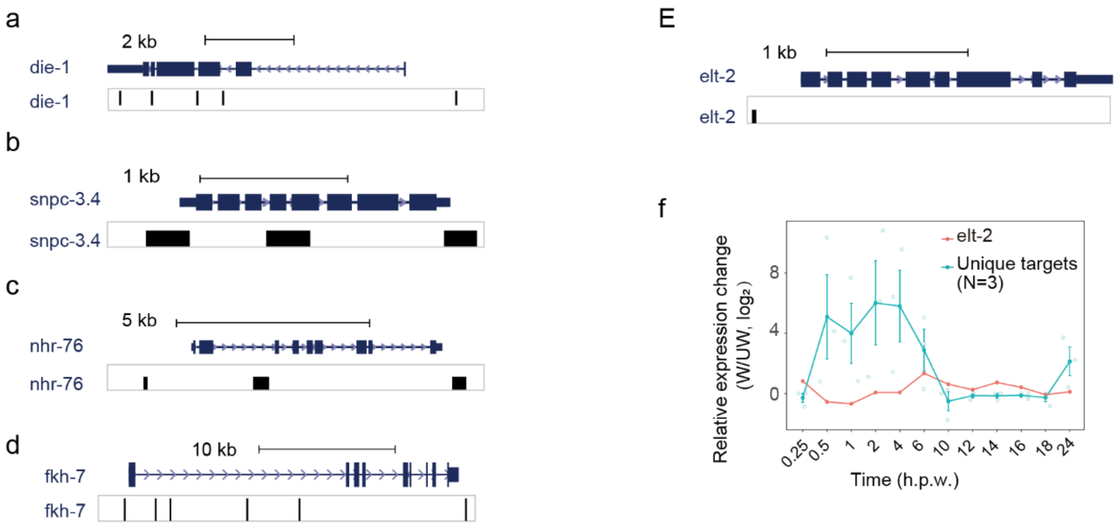

Figure S7. Putative auto-regulatory TFs at the response stage, Related to Figure 4. (**a-e**) UCSC genome browser view showing TF binding sites in the promoter regions of its own gene locus, including TF *die-1* (**a**), *snpc-3.4* (**b**), *nhr-76* (**c**), *fkh-7* (**d**), and *elt-2* (**e**). (**f**) Expression profiles of the TF *elt-2* and its potentially unique targets during wound repair.

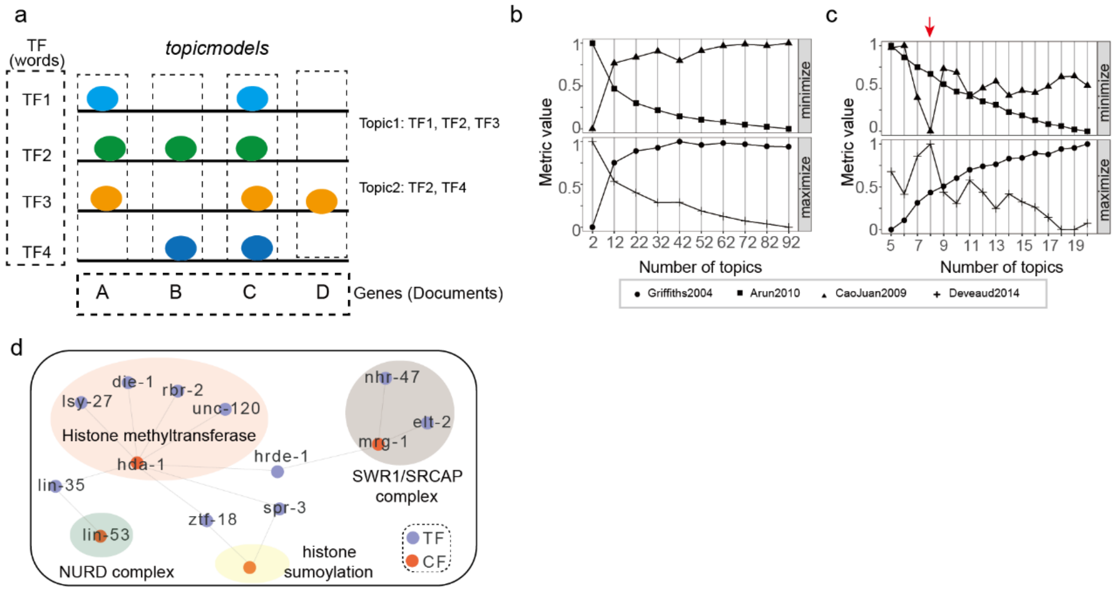

Figure S8. TF combinatorial regulation analysis by TF topic modeling, Related to Figure 5.

(**a**) Graphical illustration of the strategy of TF topic modeling in program topicmodels. (**b-c**) Computational search for the optimal number of TF topics in the range of 1-100 (**b**) and of 5-20 (**c**) based on four metrics. The red arrow highlights the best topic number 8. (**d**) Protein-protein interactions between differentially expressed TFs (blue) at the response stage and known chromatin factors (orange) in *C. elegans*.

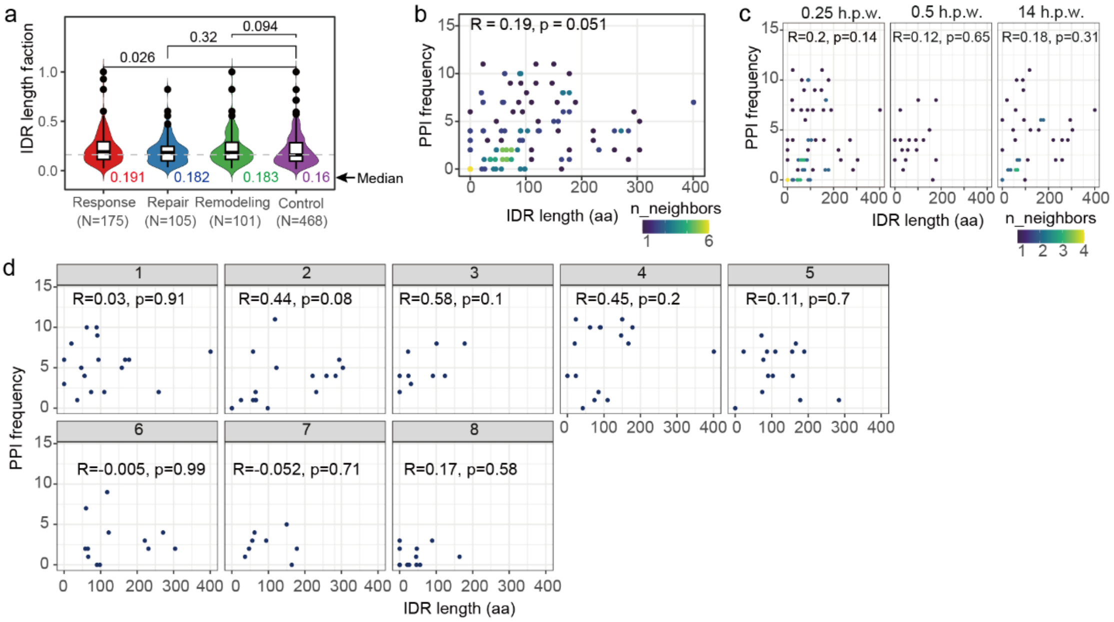

Figure S9. Relationship between IDR length and PPI, Related to Figure 5.

(**a**) Violin plot showing IDR length fraction in total protein length for TFs with IDR(s). The median IDR fractions in each group are labeled. The p-value was calculated by a one-sided Wilcoxon rank-sum test. The central line of the box plot represents the median value, and the lower and upper whiskers of the box represent the first and third quartiles, respectively. The upper whisker extends from the hinge to the largest value no further than 1.5 * IQR from the hinge (where IQR is the inter-quartile range). The lower whisker extends from the hinge to the smallest value at most 1.5 * IQR of the hinge. (**b-d**) Scatter plot showing the relationship between IDR length and protein-protein interaction (PPI) frequency of all activated TFs (**b**), TFs grouped by time points (**c**), and TFs grouped by TF topics (**d**).

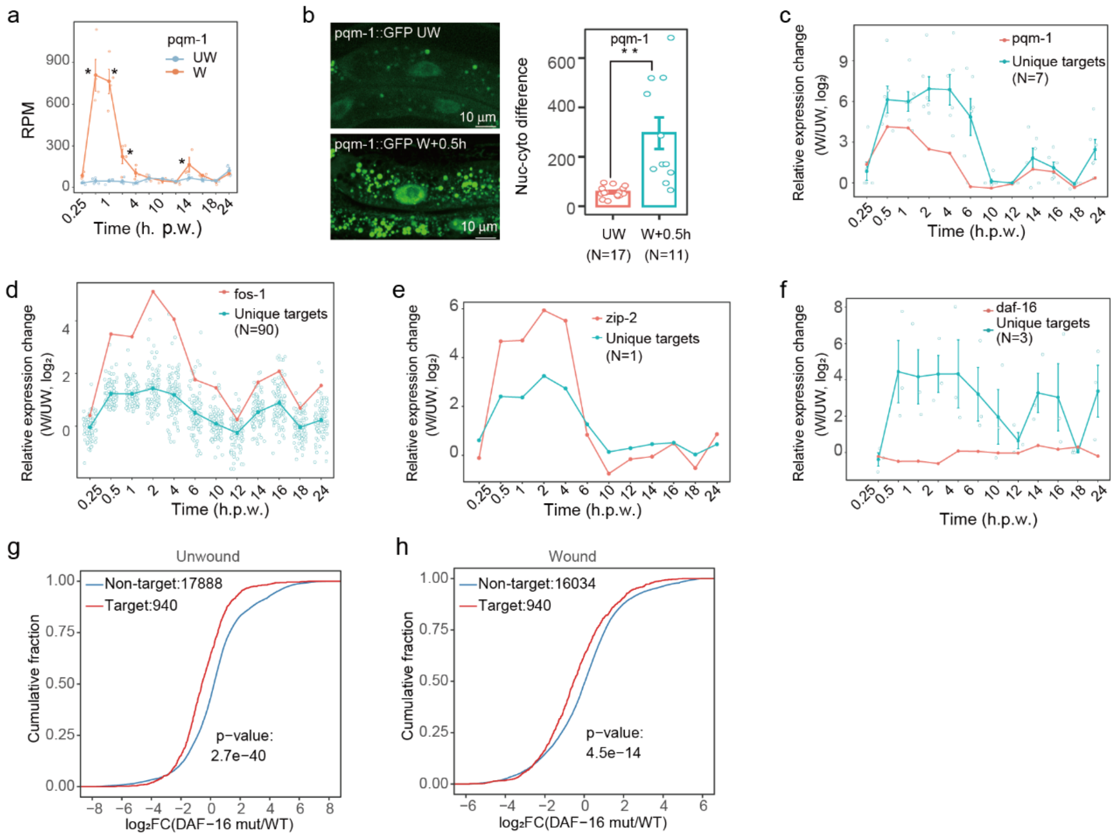

Figure S10. Expression profiles of validated TFs and their potentially unique targets, Related to Figure 7.

(a) Dynamic expression profile of pqm-1 under unwounded (UW) and wounded (W) conditions. (**b**) Fluorescence confocal images showing PQM-1 translocated into the nucleus at 0.5 hours post wounding (left) and the quantified Nuc-cyto relative fluorescence intensity (nucleus-cytoplasm) of PQM-1 in UW and 0.5 hours post wounding conditions (right). (**c-f**) Expression profiles of the TFs *pqm-1* (**c**), *fos-1* (**d**), *zip-2* (**e**), and *daf-16* (**f**) and their unique targets during wound repair. (**g-h**) Expression changes of DAF-16 putative targets under unwounded (**g**) and wounded conditions (**h**). Potential targets are from our regulatory network. The *p*-value was calculated by a one-sided Wilcoxon rank-sum test.

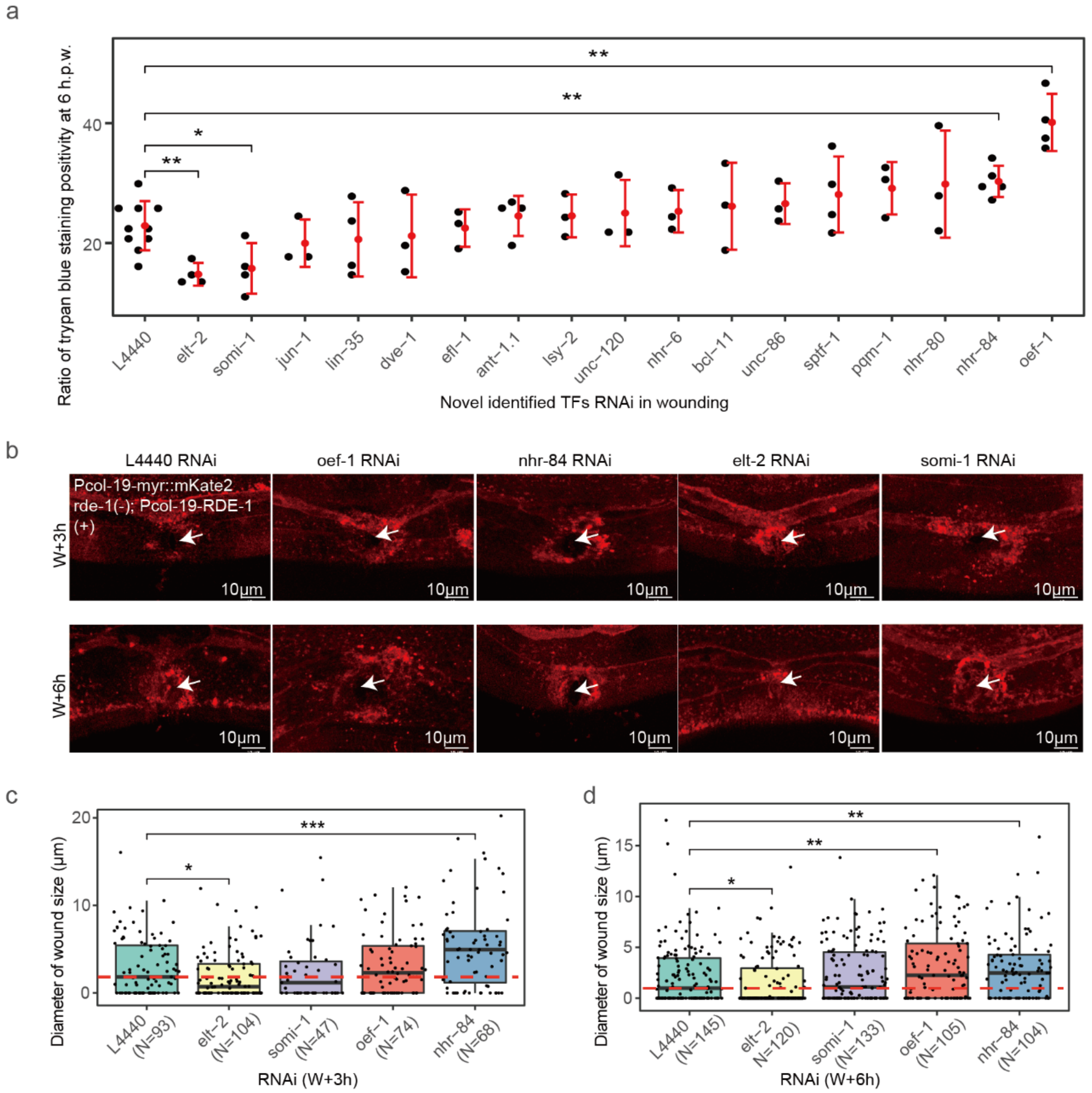

Figure S11. Validation of additional 18 novel transcription factors. Related to Figure 7. (**a**) Quantification of TryB positive staining of wounded worms at 6 h.p.w. Black points represent the ratio of positive trypan bule staining in each experiment, and red points represent mean. The error bars represent standard error. The p-value was calculated by a one-sided Wilcoxon rank-sum test. (**b**) Representative confocal images of the wounded membrane in *L4440, elt-2*, *somi-1*, *oef-1,* and *nhr-84* RNAi animals at 3 h.p.w. (top) and 6 h.p.w. (bottom). The epidermal-specific RNAi strain P*col19*-myr::mKate2;*rde-1*(-);P*col-19*-RDE-1 was used for RNAi treatment and needle wounding. White arrows indicate wound sites. Scale bars, 10 μm. (**c-d**) Quantified diameters of wound sizes in *L4440*, *elt-2*, *somi-1*, *oef-1,* and *nhr-84* RNAi animals at 3 h.p.w. (**c**) and 6 h.p.w. (**d**). The p-value was calculated by a one-sided Wilcoxon rank-sum test. * $p < 0.05$, ** $p < 0.01$, *** $p < 0.001$. The central line of the box plot represents the median value, and the lower and upper whiskers of the box represent the first and third quartiles, respectively. The upper whisker extends from the hinge to the largest value no further than 1.5 * IQR from the hinge (where IQR is the inter-quartile

range). The lower whisker extends from the hinge to the smallest value at most 1.5 * IQR of the hinge.
