# Supplementary material for: Time-course swRNA-seq uncovers a hierarchical gene regulatory network in controlling the response-repair-remodeling after wounding
Source: Commun Biol. 2024 Jun 6;7:694. doi: 10.1038/s42003-024-06352-w (PMC11156874; doi:10.1038/s42003-024-06352-w)
Supplement: Supplementary file 3 — Description of additional supplementary files [file 42003_2024_6352_MOESM3_ESM.docx]

Description of Additional Supplementary Files

**File name:** Supplementary Data 1

**Description:** Statistics of single-worm RNA-seq data.

**File name:** Supplementary Data 2

**Description:** Differentially expressed genes including iDEGs, hiDEGs, and wound-induced genes.

**File name:** Supplementary Data 3

**Description:** TF regulatory network from iDREM.

**File name:** Supplementary Data 4

**Description:** De-novo motif discovery in the promoter sequences of up-regulated genes.

**File name:** Supplementary Data 5

**Description:** Transcriptional regulatory modules including TF-topic and target-topic assignments.

**File name:** Supplementary Data 6

**Description:** Predicted IDR regions in TFs of regulatory modules.

**File name:** Supplementary Data 7

**Description:** Strains used in this study.
